# 2,2′,2′′,2′′′-(1,4-Phenyl­enedinitrilo)­tetra­acetic acid dihydrate

**DOI:** 10.1107/S1600536812019095

**Published:** 2012-05-05

**Authors:** Juanzhi Yan, Ling Ma, Miaoli Zhu, Xiangdong Zhang, Chunhua Ge

**Affiliations:** a Institute of Molecular Science, Chemical Biology and Molecular Engineering Laboratory of the Education Ministry, Shanxi University, Taiyuan, Shanxi 030006, People’s Republic of China; b Education Institute of Taiyuan University, Taiyuan, Shanxi 030001, People’s Republic of China; c Department of Biochemistry and Molecular Biology, Shanxi Medical University, Taiyuan, Shanxi 030001, People’s Republic of China; d College of Chemistry, Liaoning University, Shengyang 110036, People’s Republic of China

## Abstract

In the title compound, C_14_H_16_N_2_O_8_·2H_2_O, the complete organic molecule is generated by crystallographic inversion symmetry. The dihedral angles between the aniline ring and the acetic acid groups are almost identical, *viz.* 82.61 (7) and 80.33 (7)°. In the crystal, O—H⋯O hydrogen bonds link the organic mol­ecules and water mol­ecules, forming zigzag chains the *c* axis. An intra­molecular O—H⋯O hydrogen bond is also observed.

## Related literature

For the crystal structures of metal complexes of the title compound, see: González *et al.* (1997 ▶); Hao, Li, Chen & Zhang (2006 ▶); Hao, Li, Chen, Zhang *et al.* (2006 ▶ ▶); Zhang *et al.* (2007 ▶). For synthetic details, see: Zhang *et al.* (2007 ▶).
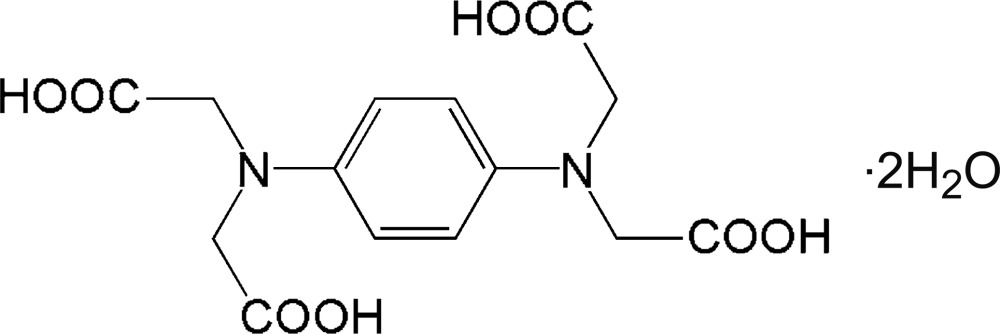


## Experimental

### 

#### Crystal data

C_14_H_16_N_2_O_8_·2H_2_O
*M* *_r_* = 376.32
Triclinic, 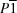

*a* = 5.1446 (12) Å
*b* = 8.4165 (19) Å
*c* = 9.953 (2) Å
α = 76.656 (4)°
β = 88.177 (4)°
γ = 85.715 (4)°
*V* = 418.12 (16) Å^3^
*Z* = 1
Mo *K*α radiation
μ = 0.13 mm^−1^
*T* = 298 K
0.20 × 0.20 × 0.20 mm

#### Data collection

Bruker SMART 1K CCD area-detector diffractometer
Absorption correction: multi-scan (*SADABS*; Sheldrick, 2000 ▶) *T*_min_ = 0.975, *T*_max_ = 0.975
2170 measured reflections
1458 independent reflections
1136 reflections with *I* > 2σ(*I*)
*R*_int_ = 0.014

#### Refinement

*R*[*F* ^2^ > 2σ(*F* ^2^)] = 0.046
*wR*(*F* ^2^) = 0.119
*S* = 1.04
1458 reflections
120 parameters
H-atom parameters constrained
Δρ_max_ = 0.15 e Å^−3^
Δρ_min_ = −0.21 e Å^−3^

### 

Data collection: *SMART* (Bruker, 2000 ▶); cell refinement: *SAINT* (Bruker, 2000 ▶); data reduction: *SAINT*; program(s) used to solve structure: *SHELXS97* (Sheldrick, 2008 ▶); program(s) used to refine structure: *SHELXL97* (Sheldrick, 2008 ▶); molecular graphics: *ORTEP-3* (Farrugia, 1997 ▶) and *SHELXL97*; software used to prepare material for publication: *publCIF* (Westrip, 2010 ▶).

## Supplementary Material

Crystal structure: contains datablock(s) I, global. DOI: 10.1107/S1600536812019095/wn2473sup1.cif

Structure factors: contains datablock(s) I. DOI: 10.1107/S1600536812019095/wn2473Isup2.hkl

Supplementary material file. DOI: 10.1107/S1600536812019095/wn2473Isup3.cml

Additional supplementary materials:  crystallographic information; 3D view; checkCIF report

## Figures and Tables

**Table 1:** Hydrogen-bond geometry (Å, °)

*D*—H⋯*A*	*D*—H	H⋯*A*	*D*⋯*A*	*D*—H⋯*A*
O5—H5*B*⋯O2^i^	0.86	2.13	2.819 (3)	137
O5—H5*A*⋯O2^ii^	0.92	2.25	3.035 (3)	143
O4—H4⋯O5	0.82	1.78	2.597 (3)	171
O1—H1⋯O3	0.82	1.86	2.653 (2)	164

Symmetry codes: (i) 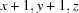; (ii) 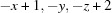.

**Fig. 1.**
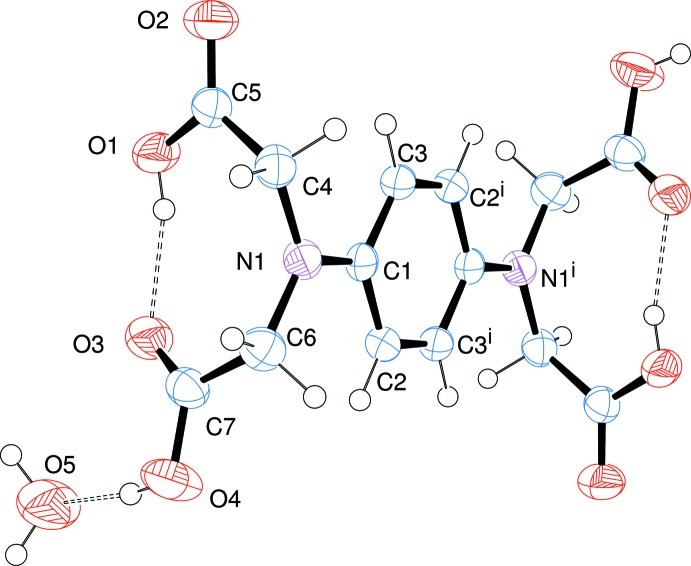
The structure of the organic molecule and one independent water molecule, showing displacement ellipsoids drawn at the 30% probability level. Hydrogen atoms are shown as spheres of arbitrary radius. The dashed lines represent hydrogen bonds. Symmetry code: i) 1 -x, -y, 1 - z.

**Fig. 2.**
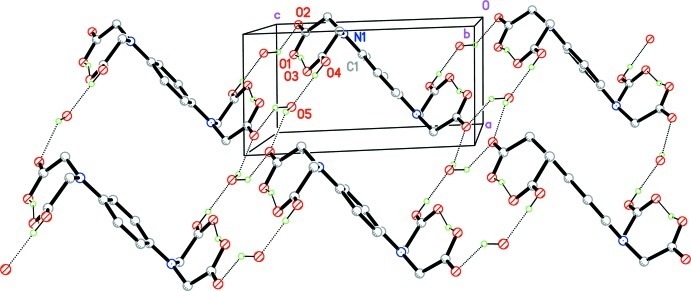
The packing of the crystal structure, showing the zigzag chains and a wave-like layer formed by O—H···O hydrogen bonds (dotted lines). H atoms not involved in the hydrogen bond interactions have been omitted for clarity.

## supplementary crystallographic information

### Comment

In recent research, the 2,2',2'',2'''-(1,4-phenylenebis(azanetriyl))tetraacetic acid ligand (H_4_dbta) formed metal complexes.(González *et al.*, 1997; Hao,Li, Chen, Zhang *et al.*, 2006; Hao, Li, Chen, & Zhang, 2006; Zhang,*et al.*, 2007). We expected to synthesize zinc complexes by the reaction of H_4_dbta with zinc chloride in water. However, colorless crystals of the title compound were obtained by evaporation of the solvent.

The crystal structure of the title compound, C_14_H_16_N_2_O_8_.2H_2_O, is centrosymmetric. The structure of the complete organic molecule and on independent water molecule are shown in Fig. 1. The dihedral angle between the plane C1/C2/C3/N1 and the plane C4/C5/O1/O2 is 82.61 (7)°, that between the plane C1/C2/C3/N1 and the plane C6/C7/O3/O4 is 80.33 (7)°.

O—H···O hydrogen bonds link the organic molecules and water molecules, forming zigzag chains (Fig. 2) An intramolecular O—H···O hydrogen bond is also observed (Fig. 1 and Table 1).

### Experimental

All reagents were of analytical grade and used without further purification. 2,2',2'',2'''-(1,4-Phenylenebis(azanetriyl))tetraacetic acid(H_4_dbta) was synthesized by a previously reported method (Zhang *et al.*, 2007). H_4_dbta (0.17 g,0.5 mmol) and ZnCl_2_ (0.136 g, 1.0 mmol) were mixed in 15 mL of water. The pH value of the solution was adjusted to 1.0 by HCl. The clear solution was allowed to stand at 323 K for 8 h. It was then filtered and the filtrate was kept at 277 K , allowing slow evaporation. After several weeks, purple single crystals of the title compound were obtained. Yield: 28%.

Selected IR(KBr, cm^-1^): 3455(*s*), 1723(*s*), 1656(*s*), 1527(*s*), 1446(*m*),1367(*s*), 1320(*m*), 1252(*m*), 1230(*m*), 1190(*m*), 972(*m*), 888(*m*), 806(*m*),736(*m*). The infrared spectra of the title compound near 3455 cm^-1^ for O—H stretching frequency showed that water of solvation existed in the crystal structure. The strong band at 1723 cm^-1^ corresponds to the C=O stretching frequency of the carboxyl group.

### Refinement

H atoms attached to C and O (carboxyl) were placed in geometrically idealized positions with C*sp*^2^—H = 0.93 Å, C*sp*^3^—H = 0.97 Å and refined in the riding model approximation; U_iso_(H) = xU_eq_(C,O), where x = 1.5 for O—H and 1.2 for C—H.

The water H atoms were located in difference Fourier maps (O—H = 0.92 and 0.86 Å) and refined, as riding, with U_iso_(H) = 1.5U_eq_(O).

### Crystal data

**Table:**

C_14_H_16_N_2_O_8_·2H_2_O	*Z* = 1
*M**_r_* = 376.32	*F*(000) = 198
Triclinic, *P*1	*D*_x_ = 1.495 Mg m^−^^3^
Hall symbol: -P 1	Mo *K*α radiation, λ = 0.71073 Å
*a* = 5.1446 (12) Å	Cell parameters from 694 reflections
*b* = 8.4165 (19) Å	θ = 2.5–25.4°
*c* = 9.953 (2) Å	µ = 0.13 mm^−^^1^
α = 76.656 (4)°	*T* = 298 K
β = 88.177 (4)°	Block, purple
γ = 85.715 (4)°	0.20 × 0.20 × 0.20 mm
*V* = 418.12 (16) Å^3^	

### Data collection

**Table:**

Bruker SMART 1K CCD area-detector diffractometer	1458 independent reflections
Radiation source: fine-focus sealed tube	1136 reflections with *I* > 2σ(*I*)
Graphite monochromator	*R*_int_ = 0.014
ω scans	θ_max_ = 25.0°, θ_min_ = 2.1°
Absorption correction: multi-scan (*SADABS*; Sheldrick, 2000)	*h* = −5→6
*T*_min_ = 0.975, *T*_max_ = 0.975	*k* = −9→9
2170 measured reflections	*l* = −7→11

### Refinement

**Table:**

Refinement on *F*^2^	Primary atom site location: structure-invariant direct methods
Least-squares matrix: full	Secondary atom site location: difference Fourier map
*R*[*F*^2^ > 2σ(*F*^2^)] = 0.046	Hydrogen site location: inferred from neighbouring sites
*wR*(*F*^2^) = 0.119	H-atom parameters constrained
*S* = 1.04	*w* = 1/[σ^2^(*F*_o_^2^) + (0.0562*P*)^2^ + 0.147*P*] where *P* = (*F*_o_^2^ + 2*F*_c_^2^)/3
1458 reflections	(Δ/σ)_max_ < 0.001
120 parameters	Δρ_max_ = 0.15 e Å^−^^3^
0 restraints	Δρ_min_ = −0.21 e Å^−^^3^

### Special details

**Table:**

Geometry. All e.s.d.'s (except the e.s.d. in the dihedral angle between two l.s. planes) are estimated using the full covariance matrix. The cell e.s.d.'s are taken into account individually in the estimation of e.s.d.'s in distances, angles and torsion angles; correlations between e.s.d.'s in cell parameters are only used when they are defined by crystal symmetry. An approximate (isotropic) treatment of cell e.s.d.'s is used for estimating e.s.d.'s involving l.s. planes.
Refinement. Refinement of *F*^2^ against ALL reflections. The weighted *R*-factor *wR* and goodness of fit *S* are based on *F*^2^, conventional *R*-factors *R* are based on *F*, with *F* set to zero for negative *F*^2^. The threshold expression of *F*^2^ > σ(*F*^2^) is used only for calculating *R*-factors(gt) *etc*. and is not relevant to the choice of reflections for refinement. *R*-factors based on *F*^2^ are statistically about twice as large as those based on *F*, and *R*- factors based on ALL data will be even larger.

### Fractional atomic coordinates and isotropic or equivalent isotropic displacement parameters (Å^2^)

**Table:**

	*x*	*y*	*z*	*U*_iso_*/*U*_eq_	
C1	0.2990 (4)	0.0540 (2)	0.58341 (19)	0.0294 (5)	
C2	0.4610 (4)	0.1632 (2)	0.5005 (2)	0.0326 (5)	
H2	0.4370	0.2740	0.4998	0.039*	
C3	0.3429 (4)	−0.1104 (3)	0.5810 (2)	0.0322 (5)	
H3	0.2384	−0.1866	0.6353	0.039*	
C4	−0.0514 (4)	−0.0116 (3)	0.7566 (2)	0.0357 (5)	
H4A	−0.1229	−0.0793	0.7021	0.043*	
H4B	−0.1969	0.0462	0.7934	0.043*	
C5	0.0940 (4)	−0.1225 (3)	0.8765 (2)	0.0361 (5)	
C6	0.0618 (4)	0.2780 (3)	0.6670 (2)	0.0389 (5)	
H6A	−0.1036	0.2976	0.7121	0.047*	
H6B	0.0541	0.3419	0.5725	0.047*	
C7	0.2789 (5)	0.3343 (3)	0.7411 (2)	0.0383 (5)	
N1	0.1002 (3)	0.1069 (2)	0.66661 (17)	0.0324 (4)	
O1	0.2911 (3)	−0.0650 (2)	0.92636 (16)	0.0483 (5)	
H1	0.3127	0.0279	0.8814	0.072*	
O2	0.0295 (4)	−0.2586 (2)	0.92856 (18)	0.0547 (5)	
O3	0.4170 (3)	0.2397 (2)	0.82357 (17)	0.0483 (5)	
O4	0.3047 (4)	0.4915 (2)	0.7071 (2)	0.0627 (6)	
H4	0.4199	0.5141	0.7532	0.094*	
O5	0.6861 (4)	0.5278 (3)	0.8597 (2)	0.0822 (7)	
H5A	0.6992	0.4518	0.9430	0.123*	
H5B	0.7121	0.6234	0.8727	0.123*	

### Atomic displacement parameters (Å^2^)

**Table:**

	*U*^11^	*U*^22^	*U*^33^	*U*^12^	*U*^13^	*U*^23^
C1	0.0321 (11)	0.0326 (11)	0.0241 (10)	−0.0036 (9)	−0.0052 (8)	−0.0065 (8)
C2	0.0418 (13)	0.0264 (11)	0.0300 (11)	−0.0033 (9)	−0.0028 (9)	−0.0069 (8)
C3	0.0368 (12)	0.0321 (11)	0.0280 (11)	−0.0099 (9)	−0.0011 (9)	−0.0052 (8)
C4	0.0328 (12)	0.0411 (13)	0.0347 (12)	−0.0073 (10)	0.0016 (9)	−0.0110 (10)
C5	0.0380 (13)	0.0375 (13)	0.0341 (12)	−0.0078 (10)	0.0018 (9)	−0.0097 (9)
C6	0.0370 (13)	0.0352 (12)	0.0437 (13)	0.0019 (10)	−0.0030 (10)	−0.0088 (10)
C7	0.0406 (13)	0.0338 (12)	0.0429 (13)	−0.0051 (10)	0.0081 (10)	−0.0139 (10)
N1	0.0328 (10)	0.0331 (10)	0.0321 (9)	−0.0034 (7)	0.0001 (7)	−0.0092 (7)
O1	0.0570 (11)	0.0431 (10)	0.0418 (9)	−0.0155 (8)	−0.0174 (8)	0.0026 (7)
O2	0.0653 (12)	0.0403 (10)	0.0558 (11)	−0.0186 (9)	−0.0060 (9)	−0.0004 (8)
O3	0.0539 (11)	0.0426 (10)	0.0487 (10)	−0.0109 (8)	−0.0130 (8)	−0.0069 (8)
O4	0.0733 (15)	0.0322 (10)	0.0833 (15)	−0.0093 (9)	−0.0134 (11)	−0.0115 (9)
O5	0.0968 (17)	0.0633 (14)	0.0892 (16)	−0.0345 (12)	−0.0230 (13)	−0.0107 (11)

### Geometric parameters (Å, º)

**Table:**

C1—C3	1.391 (3)	C5—O1	1.313 (3)
C1—C2	1.393 (3)	C6—N1	1.440 (3)
C1—N1	1.406 (3)	C6—C7	1.519 (3)
C2—C3^i^	1.385 (3)	C6—H6A	0.9700
C2—H2	0.9300	C6—H6B	0.9700
C3—C2^i^	1.385 (3)	C7—O3	1.210 (3)
C3—H3	0.9300	C7—O4	1.304 (3)
C4—N1	1.437 (3)	O1—H1	0.8200
C4—C5	1.514 (3)	O4—H4	0.8200
C4—H4A	0.9700	O5—H5A	0.9236
C4—H4B	0.9700	O5—H5B	0.8646
C5—O2	1.209 (3)		
			
C3—C1—C2	117.08 (19)	O1—C5—C4	117.95 (19)
C3—C1—N1	121.21 (19)	N1—C6—C7	111.98 (18)
C2—C1—N1	121.71 (18)	N1—C6—H6A	109.2
C3^i^—C2—C1	121.50 (19)	C7—C6—H6A	109.2
C3^i^—C2—H2	119.3	N1—C6—H6B	109.2
C1—C2—H2	119.3	C7—C6—H6B	109.2
C2^i^—C3—C1	121.4 (2)	H6A—C6—H6B	107.9
C2^i^—C3—H3	119.3	O3—C7—O4	123.3 (2)
C1—C3—H3	119.3	O3—C7—C6	122.2 (2)
N1—C4—C5	115.57 (18)	O4—C7—C6	114.4 (2)
N1—C4—H4A	108.4	C1—N1—C4	119.59 (17)
C5—C4—H4A	108.4	C1—N1—C6	119.60 (17)
N1—C4—H4B	108.4	C4—N1—C6	120.69 (17)
C5—C4—H4B	108.4	C5—O1—H1	109.5
H4A—C4—H4B	107.5	C7—O4—H4	109.5
O2—C5—O1	120.0 (2)	H5A—O5—H5B	109.1
O2—C5—C4	122.0 (2)		
			
C3—C1—C2—C3^i^	0.1 (3)	C3—C1—N1—C4	3.5 (3)
N1—C1—C2—C3^i^	179.81 (18)	C2—C1—N1—C4	−176.25 (18)
C2—C1—C3—C2^i^	−0.1 (3)	C3—C1—N1—C6	179.58 (18)
N1—C1—C3—C2^i^	−179.81 (18)	C2—C1—N1—C6	−0.1 (3)
N1—C4—C5—O2	−152.7 (2)	C5—C4—N1—C1	68.0 (2)
N1—C4—C5—O1	29.3 (3)	C5—C4—N1—C6	−108.1 (2)
N1—C6—C7—O3	−20.9 (3)	C7—C6—N1—C1	−71.6 (2)
N1—C6—C7—O4	158.3 (2)	C7—C6—N1—C4	104.5 (2)

Symmetry code: (i) −*x*+1, −*y*, −*z*+1.

### Hydrogen-bond geometry (Å, º)

**Table:**

*D*—H···*A*	*D*—H	H···*A*	*D*···*A*	*D*—H···*A*
O5—H5*B*···O2^ii^	0.86	2.13	2.819 (3)	137
O5—H5*A*···O2^iii^	0.92	2.25	3.035 (3)	143
O4—H4···O5	0.82	1.78	2.597 (3)	171
O1—H1···O3	0.82	1.86	2.653 (2)	164

Symmetry codes: (ii) *x*+1, *y*+1, *z*; (iii) −*x*+1, −*y*, −*z*+2.
